# Graded Resistance Exercise And Type 2 Diabetes in Older adults (The GREAT2DO study): methods and baseline cohort characteristics of a randomized controlled trial

**DOI:** 10.1186/s13063-015-1037-y

**Published:** 2015-11-10

**Authors:** Kylie A. Simpson, Yorgi Mavros, Shelley Kay, Jacinda Meiklejohn, Nathan de Vos, Yi Wang, Qianyu Guo, Renru Zhao, Mike Climstein, Bernard T. Baune, Steven Blair, Anthony J. O’Sullivan, David Simar, Nalin Singh, Maria A. Fiatarone Singh

**Affiliations:** Faculty of Health Science, Exercise, Health and Performance Faculty Research Group, University of Sydney, 75 East St, Lidcombe, NSW 2750 Australia; The Center for STRONG Medicine, Balmain Hospital, 29 Booth St, Balmain, NSW 2041 Australia; San Francisco, Diabetes Center, University of California, Box 0540, 513 Parnassus Ave 1119, San Francisco, CA 94143-0540 USA; Discipline of Psychiatry, The University of Adelaide, Level 4, Eleanor Harrold Building, Royal Adelaide Hospital, Adelaide, SA 5005 Australia; Department of Exercise Science, Public Health Research Building, University of South Carolina, 921 Assembly St, Columbia, SC 29208 USA; Department of Medicine, University of New South Wales, St George and Sutherland Clinical School, St George Hospital, Gray St, Kogarah, NSW 2217 Australia; Faculty of Medicine, Metabolic Disorders Research Group, University of New South Wales, Sydney, NSW 2052 Australia; Sydney Medical School, University of Sydney, Sydney, NSW 2000 Australia; Hebrew SeniorLife and Jean Mayer USDA Human Nutrition Center on Aging, Tufts University, Boston, MA USA

**Keywords:** Type 2 diabetes, Resistance training, Weight lifting, Randomized controlled trial, Power training

## Abstract

**Background:**

Type 2 diabetes (T2D) is projected to affect 439 million people by 2030. Medical management focuses on controlling blood glucose levels pharmacologically in a disease that is closely related to lifestyle factors such as diet and inactivity. Physical activity guidelines include aerobic exercise at intensities or volumes potentially unreachable for older adults limited by many co-morbidities. We aim to show for the first time the efficacy of a novel exercise modality, power training (high-velocity, high-intensity progressive resistance training or PRT), in older adults with T2D as a means for improving glycemic control and targeting many associated metabolic and physiological outcomes.

Eligibility criteria included community-dwelling men and women previously diagnosed with T2D who met the current definition of metabolic syndrome according to the International Diabetes Federation. Participants were randomized to a fully supervised power training intervention or sham exercise control group for 12 months. Intervention group participants performed whole body machine-based power training at 80%1RM, 3 days per week. The control group undertook the same volume of non-progressive, low-intensity training. Participants were assessed at baseline, 6 months and 12 months and followed for a further 5 years, during which time participants were advised to exercise at moderate-high intensity. Glycemic control (HbA1c) and insulin resistance as measured by the homeostatic model assessment 2 (HOMA2-IR) were the primary outcomes of the trial. Outcome assessors were blinded to group assignment and participants were blinded to the investigators’ hypothesis regarding the most effective intervention.

**Results:**

We recruited 103 participants (48.5 % women, 71.6 ± 5.6 years). Participants had 5.1 ± 1.8 chronic diseases, had been diagnosed with T2D for 8 ± 6 years and had a body mass index (BMI) of 31.6 ± 4.0 kg/m^2^. Fasting glucose and insulin were 7.3 ± 2.4 mmol/L and 10.6 ± 6.3 mU/L, respectively. HbA1c was 54 ± 12 mmol/mol. Eighty-six participants completed the 12-month assessment and follow-up is ongoing. This cohort had a lower-than-expected dropout (*n* = 14, 14 %) over the 12-month intervention period.

**Conclusions:**

Power training may be a feasible adjunctive therapy for improving glycemic control for the growing epidemic of T2D in older adults.

**Trial registration:**

Australian and New Zealand Clinical Trials Registry ACTRN12606000436572 (24 September 2006).

## Background

Physical activity is a critical component of optimal management for the increasing epidemic of type 2 diabetes (T2D) [1]. Although not as well studied as aerobic exercise in this cohort [2], progressive resistance training (PRT) has been shown to improve glucose homeostasis in T2D, with a clinically relevant effect size (ES) not significantly different from aerobic exercise or combined exercise or lifestyle programs [3]. It may also improve blood pressure, dyslipidemia, markers of inflammation and catabolism, and visceral obesity, thus addressing many components of metabolic syndrome [4–6]. Additionally, PRT has beneficial effects on functional exercise capacity [7, 8], osteoarthritis [9], bone health [10], depression and insomnia [11], and cognitive impairment [12], thus addressing many common co-morbidities in older adults with T2D and obesity. Importantly, PRT, in contrast to aerobic exercise, attenuates or prevents the loss of lean tissue accompanying dietary weight loss [13], thus addressing the potential adverse metabolic and clinical effects such a loss of muscle and bone mass may produce [14].

Resistance training is commonly recommended at moderate intensity and slow velocity for older adults [15]. By contrast, power training typically involves pushing a weight as quickly as possible during the concentric phase and slowly returning the weight to the start position during the eccentric phase [16]. As the rate of decline in power is greater than the rate of decline in strength with aging [17], power training may be particularly important to older adults, and has notably been shown to be more effective in improving physical function [18] and maintaining bone density [19] as compared to conventional slow-velocity resistance training. Power training has also been shown to improve functional tasks such as standing up from a chair, climbing stairs [20] and gait speed [21]. Moreover, power training specifically targets the type 2b muscle fiber atrophy that predominates in older adults and contributes to sarcopenia [22–24]. Power training thus has the potential to provide a broader spectrum of benefit than slow-velocity PRT, yet this alternative form of PRT has never been directly tested as a strategy to treat individuals with diabetes and metabolic syndrome.

With any new therapy, it is important to establish the overall risk/benefit ratio, rather than just focus upon the effect on the target symptom. Thus, our measures of other clinical problems in these individuals with metabolic syndrome, such as muscle weakness, immobility, disability, depression, cognitive impairment, sleep disturbance, postural and post-prandial hypotension, and cardiovascular symptoms will provide a balanced perspective on the unique benefits and risks of this therapeutic approach compared to usual care. It is also critically important to establish the utility of this intervention on its own, before combining it with other interventions such as weight loss diets, or aerobic exercise [4, 25] so that the independent contribution of power training to diabetes and metabolic syndrome can be definitively established.

### Objectives

Our specific aim was to conduct a 12-month randomized controlled trial (RCT) to test the efficacy of power training added to the usual medical care of older adults with T2D and metabolic syndrome. In addition, the long-term feasibility and benefits of this type of exercise will be assessed over 6 years of follow-up.

### Primary hypothesis

Power training will be associated with sustained improvements in insulin sensitivity and HbA1c compared to the sham exercise control group at 6-month and 12-month follow-up.

### Secondary hypotheses

1. Power training will be associated with improvements in the other components of metabolic syndrome and cardiovascular risk associated with T2D, including: increased circulating high-density lipoprotein (HDL) cholesterol, decreased total and low-density lipoprotein (LDL) cholesterol and triglyceride (TG) levels, decreased ambulatory blood pressure, increased aerobic capacity, improved heart rate variability and postural hypotension compared to the sham exercise control condition.
2. Power training will be associated with a significant reduction in visceral adiposity and intramuscular lipid and increase in regional and whole body measures of lean muscle mass, bone mineral density, function, and metabolism, as well as a shift in the anabolic milieu (decreased circulating and adipose tissue levels of inflammatory/catabolic cytokines and increased anabolic/anti-inflammatory factors) compared to the sham exercise control condition.
3. Body composition changes across the entire cohort will be related to observed metabolic/inflammatory improvements, whereas total body mass changes will not.
4. Randomization to the power training intervention as well as higher levels of participation in structured physical activity during follow-up will predict better outcomes in the above primary and secondary domains across the 6-year follow-up.

## Methods

### Recruitment

#### Sample size estimates

Hypothesized differences between the experimental and control participants for the primary outcomes drove sample size estimates of insulin resistance and HbA1c, based on published studies of PRT in diabetes/obesity [6, 26]. Largest available standard deviations were used for conservative estimates of ES. The most comprehensive meta-analysis at the time of study planning [27] indicated that control participants in enrolled exercise trials did not improve in metabolic outcomes, thus the control change was set at 0. Setting beta at 0.2, and alpha at 0.05, and n1 = n2, the following sample size requirements were estimated for changes from baseline at 12 months (Table 1).

**Table 1:** Primary outcomes and effect sizes

Primary outcome	Experimental mean change	Sham control Mean change	Pooled standard deviation	Effect size (*d*)	Sample required (total cohort)
HOMA2-IR	−0.6	0	0.8	0.75	52
HbA1c (mmol/mol)	−13.1	0	18.6	0.70	78

Sample size estimates were driven by hypothesized differences between the experimental and control participants in the primary outcomes of the trial: insulin resistance and glucose homeostasis, as measured using HOMA2-IR and HbA1c respectively. Effect sizes were calculated based on an average of published studies of PRT in diabetes/obesity [6, 26].

*HbA1c* glycosylated hemoglobin, *HOMA2*-*IR* homeostatic model of assessment of insulin resistance 2

Thus, 98 participants recruited, (assuming 20 % attrition), would provide 78 participants at 12 months, as required by the smaller ES hypothesized for HbA1c. Based on our pilot work, we estimated we would need to screen approximately 500 participants by phone, and 125 in person, to enroll approximately 100 eligible/interested participants. We anticipated that our loss to follow-up would actually be less than 20 %.

#### Recruitment strategies

Participants were recruited via general practitioner referral, targeted mail-outs, advertisements in local newspapers and seniors’ magazines and from brochures distributed to local medical practitioners and pharmacies. Participants were recruited from August 2006 to December 2010 with the final 12-month assessment in December 2011. Follow-up testing is due for completion in February 2016.

#### Management

All incoming responses were recorded by the research assistant and logged in a tracking database that outlined the flow of participants through the recruitment, screening and (if eligible) intervention process.

#### Screening

Potential participants contacted the research assistant and a telephone interview was conducted or scheduled for a convenient time. The telephone screening was comprised of questions to ascertain the participant’s basic demographic and contact information, current diabetic and health status, medical history, current medication use and physical activity/exercise levels. The study physician reviewed all of the individual screenings and participants were subsequently notified of their eligibility. If eligible, participants were requested to attend the University of Sydney for subsequent testing (including a health and physical examination by a physician and a 12-lead electrocardiogram (ECG) and maximal exercise stress test) to determine their eligibility.

### Participants

#### Eligibility criteria for participants

**Fig. 1:**
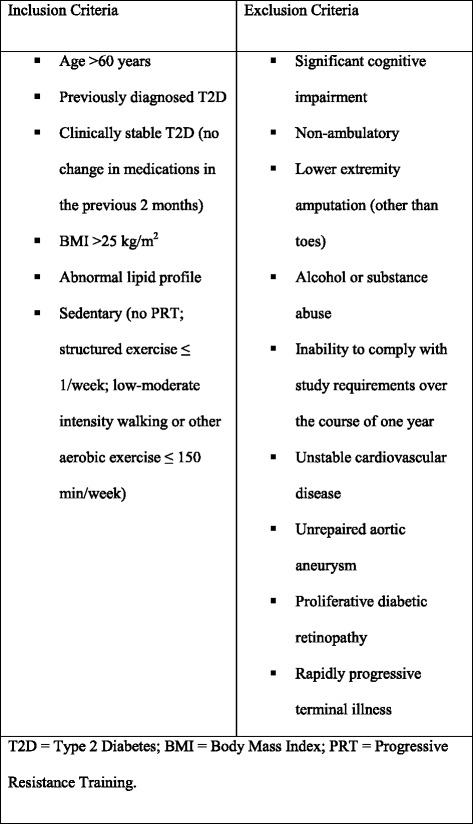
Inclusion and exclusion criteria. *BMI* body mass index, *PRT* progressive resistance training, *T2D* type 2 diabetes

The inclusion and exclusion criteria are listed in Fig. 1. Participants eligible for inclusion were treated with diet alone, oral medications or insulin or combination at the time of enrollment. Metabolic syndrome was defined according to the International Diabetes Federation as:

- Central obesity (defined as waist circumference ≥ 94 cm for Europid men and ≥ 80 cm for Europid women, plus any 2 of the following 4 factors:
- Raised TG level: ≥ 1.7 mmol/L, or specific treatment for this lipid abnormality
- Reduced HDL cholesterol: < 1.03 mmol/L) in men or < 1.29 mmol/L in women, or specific treatment for this lipid abnormality
- Raised resting blood pressure: systolic blood pressure (BP) ≥ 130 mmHg or diastolic BP ≥ 85 mmHg, or treatment of previously diagnosed hypertension
- Raised fasting plasma glucose (FPG) ≥ 5.6 mmol/L, or previously diagnosed T2D.

Exclusion criteria included significant cognitive impairment (inability to comprehend informed consent or evidence of functional impairment related to cognition on screening), non-ambulatory status or lower extremity amputation other than toes, current alcohol or substance abuse, inability to comply with study requirements over the course of 1 year due to travel plans or other commitments, or specific contraindications to resistance training exercise, such as unstable cardiovascular disease, aortic aneurysm, proliferative diabetic retinopathy, uncontrolled hypertension, or rapidly progressive or terminal illness. Temporary exclusions included recent laser or other ocular surgery (within 2 weeks), symptomatic hernias, acute illness, or recent fracture or other injury, until resolved.

#### Settings and location where data were collected

All assessments were carried out at the University of Sydney, Faculty of Health Sciences, Lidcombe and in the Radiology Department of Royal Prince Alfred Hospital, Sydney. Training sessions were conducted at the Harbord Diggers’ Freshwater Fitness Center, Harbord and at The Center for STRONG Medicine, Balmain Hospital, Balmain.

### Interventions

#### Experimental intervention: power training

Experimental participants performed power training 3 days per week and using 8 major muscle groups on pneumatic resistance under supervision. We used a version of PRT called power training, in which the concentric contraction (lifting the weight) was done as fast as possible, and the lowering of the weight was done slowly (over 2–3 seconds). The exercises targeted the majority of the large muscle groups of the arms, legs, and trunk and consisted of lateral pulldown, chest press, upper back, leg press, knee extension, and knee flexion, hip extension and hip abduction. These are symmetrical muscle groups and functionally relevant to the activities of daily living, gait, and balance of older adults. For each exercise, participants performed 3 sets of 8 repetitions with a fast concentric and slow eccentric phase on pneumatic resistance training machines (approximately 6 seconds per repetition with 2 minutes of rest between sets), a regimen which has been shown to produce optimum adaptations in terms of muscle power, strength, endurance in older adults [16].

The intensity was set at 80 % of the most recently determined peak strength (1 repetition maximum or 1RM). Resistances used were increased as tolerated using the Borg Scale Rating of Perceived Exertion [28, 29] on a continuous basis throughout the 12 months, and 1RM testing was repeated at 2-week intervals to ascertain progress and regulate intensity. All training was fully supervised by skilled exercise physiologists to maintain proper intensity and progression, as 2 separate trials in T2D have now shown that metabolic benefits of PRT disappear when participants are switched to semi-supervised community-based or home-based training [30, 31]. This was attributed to a drop in adherence and intensity when supervision was withdrawn. As this is a first-time efficacy trial of power training, our intent was to maximise protocol adherence.

#### Control group intervention: sham PRT

The same trainers supervised sham exercise control participants in the same facility but at different hours to avoid contamination and unblinding. These participants performed three sets of eight repetitions on the same machines, with no loading beyond the weight of the bar of the machine, using slow concentric and eccentric contraction speed. No interim 1RM testing and no progression took place. We have found that similar regimens do not improve muscle function or mass, functional status, mobility, depression, aerobic capacity, or other clinical outcomes we are measuring [32, 33]. Low-intensity resistance training has also been shown to have no effect on visceral fat [34], adiponectin [35], glucose homeostasis or insulin sensitivity, or bone mineral density [36], thus providing an ideal sham exercise control condition.

### Outcomes

The primary outcomes of this study were insulin resistance 96 hours after the last exercise bout as assessed by the homeostatic model assessment 2 (HOMA2) computer model and HbA1c. The 96-hour time interval for HOMA2 was chosen to minimize the well-described acute bout effect of exercise on insulin sensitivity, as we are primarily interested in long-term training adaptations related to visceral fat/muscle mass.

Secondary outcomes and covariates include all of the components of metabolic syndrome, body composition, adipokines, muscle morphology and metabolism, genetic and epigenetic markers related to metabolic/cardiovascular health and exercise adaptation, measures of energy expenditure and fat oxidation, neuropsychological function, cardiovascular health status, quality of life, dietary intake and habitual physical and sedentary activity levels. Blinded assessors, at a laboratory facility separate from the training site to prevent unblinding, repeated all measurements at baseline, 6 months, and 12 months in experimental and control participants. In addition, selected measures were repeated annually during the 5 additional years of follow-up.

#### Domains of assessment

**Fig. 2:**
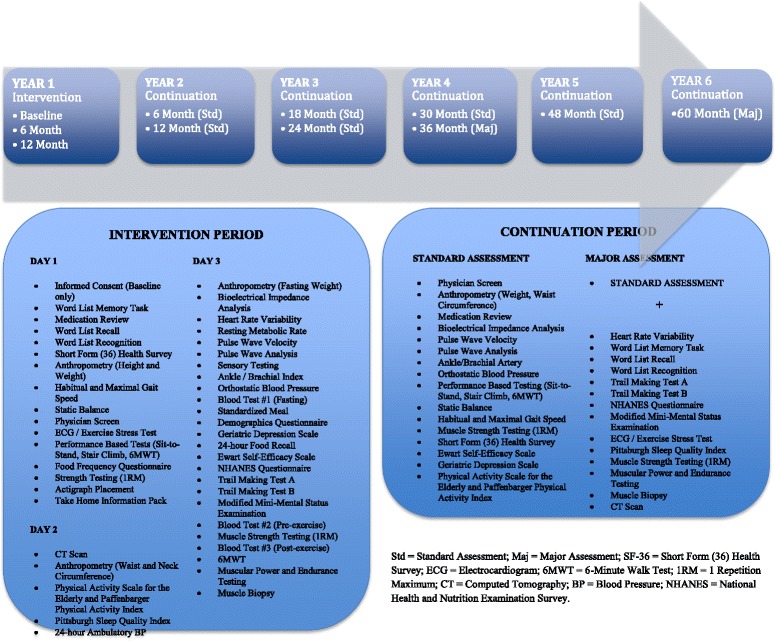
Timeline of assessments. **a** 6-month and 12-month assessments performed 72 hours after previous training session. **b** 6-month and 12-month assessments performed 48 hours after previous training session. **c** 6-month and 12-month assessments performed 96 hours after previous training session. *1RM* 1 repetition maximum, *6MWT* 6-Minute Walk Test, *BP* blood pressure, *CT* computed tomography, *ECG* electrocardiogram, *SF-36* 36-Item Short-Form Health Survey, *NHANES* National Health and Nutrition Examination Survey

All outcome measures administered by blinded assessors at baseline, 6-month and 12-month follow-up are listed in Table 2. During the intervention phase, assessments were conducted over 3 different days to allow for the correct timing of exercise bouts and blood draws and to ensure adequate participant preparation (e.g. the cessation of blood thinning medication where possible). Figure 2 details the timing of assessments throughout the course of the study.

**Table 2:** Outcome measures

Outcome	Outcome measure	Description
Primary	Insulin sensitivity	72 hours and 96 hours post exercise
		• HOMA2 computer model for insulin sensitivity (IR, %S) [53]
		• HOMA2 computer model for beta cell function (%Beta)
	Glucose homeostasis	Fasting glucose
		Glycated hemoglobin [53]
		Insulin
		C-peptide levels
		Diabetic medication inventory and dosages
		Meal tolerance test
Secondary	Cardiovascular health	Resting heart rate variability and pulse wave velocity (arterial stiffness)
		24-hour ambulatory blood pressure
		Postural blood pressure
		Ankle brachial blood pressure index
	Lipid metabolism	Total cholesterol
		Low/High-density cholesterol
		Triglycerides
		Basal fat oxidation via indirect calorimetry
		Intramuscular lipid content
	Muscle morphology and metabolism	Muscle tissue biopsy (vastus lateralis) [54]
		• Glucose transporter type 4 receptor protein
		• Intramuscular insulin-like growth factor 1
		• Glycogen content
		• Muscle fiber cross-sectional area
		• Muscle fibrertyping
	Adipokines and Inflammatory markers	Adipose tissue biopsy [54]
		• TNF-α
		• Interleukin-6
		• High molecular weight adiponectin
		• C-Jun N-terminal kinase
		• Serum C-reactive protein [55]
	Body composition [53]	Bioelectrical impedance analysis
		• Skeletal muscle mass, skeletal muscle mass index
		• Fat free mass
		• Fat mass
		• Body fat percentage
		Computed tomography scan
		• Abdominal and thigh girth, sagittal diameter
		• Abdominal total, visceral and subcutaneous fat area
		• Thigh total, intramuscular and subcutaneous fat area and muscle density (intramuscular lipid index)
		• Thigh muscle area
		Waist circumference
		Body mass index
	Exercise capacity and Functional status	Exercise stress test (maximal treadmill test with indirect calorimetry)
		• Peak aerobic capacity
		• Anaerobic threshold
		• Oxygen uptake efficiency slope
		• Heart rate recovery
		Muscle strength, power and endurance
		Habitual and maximal gait speed
		Static and dynamic balance
		Chair stand and stair climb power
	Neuropsychological profile	Geriatric Depression Scale
		Pittsburgh Sleep Quality Index
		Ewart’s Self-efficacy Scale
		Cognitive function
		• Mini Mental State Exam
		• Word List Recognition and Recall
		• Trail Making A and B
		Actigraph sleep architecture over 7 days
		• Total time in/out of bed
		• Sleep latency, sleep efficiency
		• Total time asleep
		• Number of awakenings
	Health-related quality of life	Medical Outcomes Study 36-Item Short-Form Health Survey
	Nutritional intake	Food frequency questionnaire of Bloch over past 4 months
	Energy expenditure	Resting metabolic rate via indirect calorimetry
		Habitual physical activity and sedentary behaviour via Physical Activity Scale for the Elderly
		Actigraph accelerometers over 7 days
		• Waking hours
		• Wear time
		• Total sedentary time, mean sedentary time, median sedentary time
		• Percentage of time spent in sedentary (1 MET), light (<3 MET), moderate (3–6 MET) and vigorous (>6 MET) activity
		• Total energy expenditure spent in sedentary (1 MET), light (<3 MET), moderate (3–6 MET) and vigorous (>6 MET) activity
		• Physical activity level (PAL score)

*%S* percent sensitivity, *HOMA2* homeostatic model assessment 2, *MET* metabolic equivalent of task, *TNF-α* tissue necrosis factor alpha

### Randomization

#### Sequence generation

A computerized random-number generator (http://www.randomization.com, created by Dr Gerard E. Dallal, Tufts University) was used to randomize eligible participants at the level of the individual participant, stratified by sex and use of insulin, in blocks of four.

#### Allocation concealment

Sealed envelopes were prepared by an independent researcher, containing sequential treatment assignments based on a computer-generated randomization scheme, and opened by the participant’s trainer after completion of all baseline testing.

#### Implementation

The study was approved by the University of Sydney and the Sydney South West Area Health Service Human Research Ethics Committees (RPA HREC protocol number × 04-009602/08/2006). A blinded outcomes assessor obtained informed consent from all participants enrolled in the study and informed the independent researcher when the participant was eligible for randomization (after completion of baseline testing). Participants were notified of their training session time by the exercise physiologist administering the intervention.

### Blinding

This is the first truly double-blind sham exercise-controlled RCT in T2D. All outcomes assessors were blinded to treatment assignment for the duration of the study. Only the exercise physiologists and medical practitioners responsible for administering and overseeing the exercise programs were informed of participant group assignment. Participants were also blinded to the investigators’ hypothesis and only informed of their training time, not group assignment as active or sham, and were told that two different kinds of exercise were being compared for efficacy.

### Statistical methods

All outcomes will be analyzed using all available data without imputation for missing time points via repeated measures linear mixed models, without regard to discontinuation or dropout. Secondary per-protocol analyses will be carried out on participants with ≥ 70 % compliance to exercise. All mixed models will be adjusted for relevant covariates if necessary, with group by time interaction as the primary outcome of interest. Covariates will be chosen as appropriate via a priori hypothesized confounders if there are differences between groups within relevant variables at baseline within the cohort despite randomization. Relationships between variables of interest at baseline between changes scores will be determined by simple and stepwise regression models and logistic regression models as appropriate. Statistical significance will be initially assumed at the 0.05 level, as all hypotheses were specified a priori, and Bonferroni correction is considered overly conservative in this instance [37]. Effect sizes and 95 % confidence intervals (CIs) will be calculated for all outcomes. Calculations of ES will be adjusted via Hedges’ bias-corrected ES for small sample sizes [38] and interpreted according to Cohen’s interpretation of “trivial” (<0.20), “small” (≥0.20 < 0.50), “moderate” (≥0.50 < 0.80), and “large” (≥0.80) ES [39]. Ninety-five percent CIs for the relative ES will calculated. For ES in non-normally distributed data, median will substituted for mean, and range/4 will be substituted for SD.

## Results

### Participant flow

**Fig. 3:**
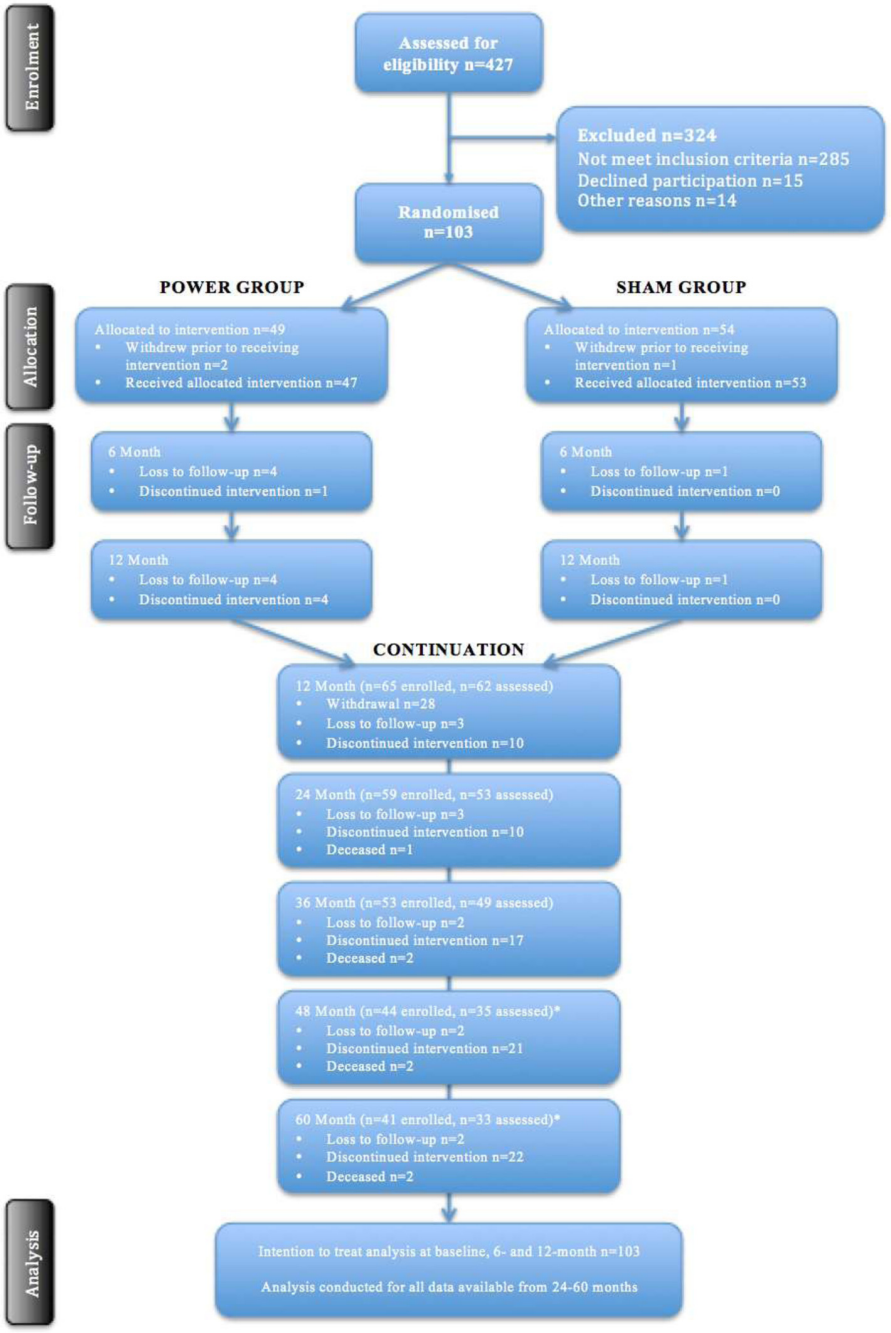
Consolidated Standards of Reporting Trials (CONSORT) flow chart, Loss to follow-up and deceased numbers are cumulative. The numbers for missed assessments are specific to each time point. A linear mixed-effects model with repeated measures will be used to determine changes over time as this method allows for all available data to be used without imputation for missing values. Thus, even with dropouts, all participants who entered the study at baseline will be entered into the model

Participants were assessed at baseline, 6 and 12 months for the intervention phase of study and annually for the remaining 5-years of follow-up. Figure 3 shows the flow of participants through the study.

### Recruitment

We assessed 427 people for eligibility (Fig. 3). After completing the telephone screening questionnaire, 324 people were deemed ineligible for not meeting the inclusion criteria (*n* = 285), declining to participate (*n* = 15), and a further 24 people were either unable to commit, had transport difficulties/did not live local to the training sites or had medical contraindications. A total of 103 people gave consent, met the eligibility criteria and were randomized to either the power training intervention group (*n* = 49) or the sham-exercise control group (*n* = 54).

### Losses and exclusions

Three participants withdrew from the study prior to commencing the intervention. Fourteen participants dropped out of the study (did not complete follow-up assessments) after commencing the intervention (intervention group *n* = 10; control group *n* = 4). One was an adverse event related to the intervention (musculoskeletal injury in the intervention group), five felt the intervention was too hard, four were medical concerns unrelated to the intervention, three were due to commitment issues, and one was due to disinterest. Our attrition of 14 % at 12 months was less than expected for the intervention period. As expected, there has been a steady decline in participation over the follow-up phase. A total of 70 people commenced the 5-year post-intervention program with a total of 41 participants expected to complete the entire 6 years of follow-up. Currently, 35 participants have completed the 6-year follow-up, with the remaining 6 participants scheduled for their final assessment.

### Baseline demographics

The baseline participant characteristics are reported in Table 3. Forty-nine and 54 participants were randomized to the invervention and control groups, respectively. Our eligibility criteria were designed so as to maximize external validity as only terminal or unstable diseases, or conditions which would permanently preclude resistance training, were exclusionary. Thus, our cohort was generally representative of older diabetic cohorts in Australia and other countries, with multiple co-morbidities and chronic medication usage. For example, the Australian Institute for Health and Welfare 2007–8 [40] report states that 58 % of individuals with diabetes also have cardiovascular disease. In our cohort, nearly one half (49 %) were being treated for cardiovascular disease. Similarly, 43 % of the Graded Resistance Exercise And Type 2 Diabetes in Older adults (GREAT2DO) cohort had hypercholesterolemia at baseline, which is not dissimilar to the Australian adult population where half of all adults over 25 years of age have high total cholesterol levels (data from 1999–2000) [41]. Among the 103 participants in this study, only 5.8 % were current smokers, similar to the 4.4 % reported in the Look AHEAD study of adults with T2D in 2006 [42]. Sixteen participants reported insulin use for control of their diabetes. In comparison to some previous trials that have excluded people with cardiovascular disease and insulin therapy, overall our cohort is more representative of the general population of older adults with T2D.

**Table 3:** Baseline participant characteristics

	Power	Sham	Total
	(*n* = 49)	(*n* = 54)	(*n* = 103)
Demographics			
Age (years)	66.9 ± 4.7	68.8 ± 6.1	67.9 ± 5.5
Sex (female)	24.0 (49.0)	27.0 ± (50.0)	51.0 ± 0.5
Smoking status			
Current	3.0 (6.1)	3.0 (5.6)	6.0 (5.8)
Past	31.0 (63.3)	33.0 (61.1)	64.0 (62.1)
Ethnic origin			
Caucasian	49.0 (100.0)	50.0 (92.6)	99.0 (96.1)
Asian	0.0 (0.0)	2.0 (3.7)	2.0 (1.9)
Indian	0.0 (0.0)	2.0 (3.7)	2.0 (1.9)
Chronic diseases			
Duration of diabetes (years)	6.9 ± 5.1	9.1 ± 6.7	8.0 ± 6.0
Number of chronic diseases	5.1 ± 1.9	5.1 ± 1.8	5.1 ± 1.8
Chronic diseases			
Hypertension	37.0 (75.5)	39.0 (72.2)	76.0 (73.8)
Osteoarthritis	35.0 (71.4)	33.0 (61.1)	68.0 (66.0)
Cardiovascular diseases	23.0 (46.9)	27.0 (50.0)	50.0 (48.5)
High cholesterol	24.0 (49.0)	21.0 (38.9)	45.0 (43.7)
Cancer	14.0 (28.6)	17.0 (31.5)	31.0 (30.1)
GERD (Reflux/Barrett’s esophagus)	8.0 (16.3)	14.0 (25.9)	22.0 (21.4)
PVD	10.0 (20.4)	11.0 (20.4)	21.0 (20.4)
IHD, MI, Angina	8.0 (16.3)	11.0 (20.4)	19.0 (18.4)
Sleep apnea	7.0 (14.3)	12.0 (22.2)	19.0 (18.4)
Depression	10.0 (20.4)	8.0 (14.8)	18.0 (17.5)
Hypothyroidism	7.0 (14.3)	9.0 (16.7)	16.0 (15.5)
Arrhythmia	8.0 (16.3)	6.0 (11.1)	14.0 (13.6)
Ulcers (gastrointestinal tract)	7.0 (14.3)	7.0 (13.0)	14.0 (13.6)
Osteoporosis/Osteopenia	5.0 (10.2)	8.0 (14.8)	13.0 (12.6)
Gout	7.0 (14.3)	5.0 (9.3)	12.0 (11.7)
Benign prostatic hyperplasia	3.0 (6.1)	8.0 (14.8)	11.0 (10.7)
Chronic venous disease	4.0 (8.2)	6.0 (11.1)	10.0 (9.7)
COPD/CAL	2.0 (4.1)	7.0 (13.0)	9.0 (8.7)
Hyperthyroidism	2.0 (4.1)	6.0 (11.1)	8.0 (7.8)
Esophagitis	5.0 (10.2)	3.0 (5.6)	8.0 (7.8)
Health status			
Weight (kg)	89.5 ± 15.2	88.8 ± 18.8	89.1 ± 17.1
BMI (kg/m^2^)	31.5 ± 4.7	31.6 ± 6.0	31.6 ± 5.4
Waist circumference (cm)			
Men	112.4 ± 9.6	109.3 ± 11.5	110.8 ± 11.7
Women	109.6 ± 9.6	108.7 ± 11.5	109.1 ± 12.6
Resting blood pressure (mmHg)			
Systolic	147.2 ± 17.9	145.1 ± 17.9	146.1 ± 17.9
Diastolic	78.9 ± 7.5	77.4 ± 10.2	78.1 ± 9.0
Resting heart rate (bpm)	65.2 ± 12.1	64.6 ± 8.4	64.9 ± 10.2
Fasting glucose (mmol/L)	7.4 ± 2.5	7.1 ± 2.2	7.3 ± 2.4
Fasting insulin (mU/L)	10.1 ± 5.9	11.1 ± 6.7	10.6 ± 6.3
HbA1c (%)	6.9 ± 0.9	7.3 ± 1.3	7.1 ± 1.1
HOMA2-IR	2.6 ± 1.0	2.9 ± 1.3	2.7 ± 1.1
Cholesterol (mmol/L)			
Total	4.5 ± 1.1	4.2 ± 1.1	4.4 ± 1.1
HDL	1.2 ± 0.3	1.2 ± 0.4	1.2 ± 0.3
LDL	2.5 ± 0.9	2.3 ± 0.9	2.4 ± 0.9
Triglycerides	1.7 ± 1.0	1.7 ± 0.9	1.7 ± 0.9
C-reactive protein (mg/L)	3.8 ± 3.2	4.5 ± 4.9	4.2 ± 4.2
Diabetic treatment			
Diet only	8.0 (16.0)	10.0 (19.0)	18.0 (17.0)
Oral hypoglycemics only	34.0 (69.0)	35.0 (65.0)	69.0 (67.0)
Oral hypoglycemics and Insulin	4.0 (8.0)	7.0 (13.0)	11.0 (11.0)
Insulin only	3.0 (6.0)	2.0 (4.0)	5.0 (5.0)
Physical Function			
Habitual gait speed (m/s)	1.2 ± 0.2	1.2 ± 0.2	1.2 ± 0.2
Maximal gait speed (m/s)	1.9 ± 0.3	1.9 ± 0.3	1.9 ± 0.3
6MWT (m)	551.1 ± 93.8	539.1 ± 95.3	544.8 ± 94.3

Values are means ± SD or *n* (%)

*6MWT* 6-Minute Walk Test, *BMI* body mass index, *BPH* benign prostatic hyperplasia, *COPD/CAL* chronic obstructive pulmonary disease/chronic airflow limitation, *GERD* gastro-esophageal reflux disease, *HbA1c* glycemic control, *HDL* high-density lipoprotein, *HOMA2-IR* homeostatic model assessment 2, *IHD* ischemic heart disease, *LDL* low-density lipoprotein, *MI* myocardial infarction, *PVD* peripheral vascular disease,

### Adverse events

There were eight adverse events in six participants adjudicated by the study physician to be related to study procedures; seven in the intervention group and one in the control group. These included three syncopal episodes in a male participant with known syncope, one hamstring strain, one back pain leading to dropout, one exacerbation of pre-existing umbilical hernia requiring surgical repair, one subscapularis tear in a female participant with pre-existing grade IV osteoarthritis/rotator cuff disease requiring surgery, and one partial thickness tear of a rotator cuff muscle managed conservatively.

## Discussion

We have shown for the first time that it is feasible to recruit and retain older adults with T2D and multiple chronic co-morbidities in a long-term trial of high-intensity power training. Few RCTs of PRT, as an isolated addition to usual care in middle-aged and older adults with T2D, have been published: however, the magnitude of change in glucose control (HbA1c) in these studies was comparable to the effect of aerobic exercise or oral hypoglycemic therapy [3]. One study, which directly compared isolated PRT to aerobic exercise in middle-aged type 2 adults with diabetes [26], found that PRT significantly improved 48-hour continuous glucose control, HbA1c, insulin sensitivity, and lipids, whereas aerobic exercise was ineffective. Limitations of previous studies of PRT include small sample sizes and relatively short periods of follow-up in some cases, and lack of comprehensive assessment of mechanisms of benefit, cardiovascular profile and associated clinical benefits relevant to older adults.

Thus, despite the strong theoretical rationale for its use in this cohort, and its recent advocacy by expert consensus panels internationally [1, 14, 43], PRT is not a common treatment for diabetes [2] and more evidence of feasibility, safety, and efficacy are needed. This need is highlighted by the results of the Look AHEAD trial, which reported no difference in the primary outcome of cardiovascular events following lifestyle modification in type 2 diabetics, despite clear benefits in secondary outcomes such as weight, physical activity level, fitness, quality of life, depression levels and metabolic risk [44–46]. However, most lifestyle interventions, even intensive ones such as Look AHEAD, may not attenuate losses of lean tissue mass seen with both aging and caloric restriction. In fact, bone losses were greater in men in the intensive lifestyle group in Look AHEAD compared to controls, and were proportional to weight loss achieved [47, 48]. This important issue of lean tissue loss accompanying lifestyle modification programs that do not prioritize or include anabolic exercises thus requires additional study, and is the focus of the GREAT2DO trial described here.

## Conclusion

To our knowledge no study investigating high-intensity power training has yet been published, and thus the GREAT2DO study will provide the first evidence of the safety, efficacy and long-term feasibility of this novel modality of anabolic exercise in older adults with T2D. Other studies suggest that power training is useful for osteoporosis [49, 50], balance [51], functional performance [18] and quality of life [52]. If our hypotheses are correct, improvement in metabolic health and other secondary outcomes from this trial may add to the growing rationale for this unique and robust form of exercise training for the treatment of chronic disease-related and age-related syndromes.

## Abbreviations

1RM: 1 repetition maximum
6MWT: 6-Minute Walk Test
BMI: body mass index
BP: blood pressure
BPH: benign prostatic hyperplasia
COPD/CAL: chronic obstructive pulmonary disease/chronic airflow limitation
CT: computed tomography
ECG: electrocardiogram
FPG: fasting plasma glucose
GERD: gastro-esophageal reflux disease
GREAT2DO: Graded Resistance Exercise And Type 2 Diabetes in Older adults
ES: effect size
HbA1c: glycemic control
HDL: high-density lipoprotein
HOMA: homeostatic model assessment
IHD: ischemic heart disease
IR: insulin resistance
LDL: low-density lipoprotein
MI: myocardial infarction
NHANES: National Health and Nutrition Examination Survey
PRT: progressive resistance training
PVD: peripheral vascular disease
RCT: randomized controlled trial
SF-36: Medical Outcomes Study 36-Item Short-Form Health Survey
T2D: type 2 diabetes
TG: triglycerides
